# Anti-proliferative activity of RIHMS-Qi-23 against MCF-7 breast cancer cell line is through inhibition of cell proliferation and senescence but not inhibition of targeted kinases

**DOI:** 10.1186/s12885-023-11547-1

**Published:** 2023-11-02

**Authors:** Randa El-Gamal, Sara Elfarrash, Mohammad EL-Nablaway, Asmaa Ahmed Salem, Seyed-Omar Zaraei, Hanan S. Anbar, Ashraf Shoma, Mohammed I. El-Gamal

**Affiliations:** 1 Department of Medical Biochemistry & Molecular Biology, Faculty of Medicine, Mansoura University, Mansoura, 35516 Egypt; 2 Medical Experimental Research Center (MERC), Faculty of Medicine, Mansoura University, Mansoura, 35516 Egypt; 3 Department of Medical Biochemistry, Faculty of Medicine, Horus University, New Damietta, Egypt; 4 Department of Medical Biochemistry, Faculty of Medicine, University of Mansoura, Mansoura, Al-Daqahlia Governorate 35516 Arab Republic of Egypt; 5 Department of Medical Physiology, Faculty of Medicine, Mansoura University, Mansoura, 35516 Egypt; 6 Department of Basic Medical Sciences, College of Medicine, AlMaarefa University, PO Box 71666, Riydah, 11597 Kingdom of Saudi Arabia; 7 Department of Clinical Oncology and Nuclear Medicine, Faculty of Medicine, Mansoura University, Mansoura, Egypt; 8 Research Institute of Medical and Health Sciences, University of Sharjah, Sharjah, 27272 United Arab Emirates; 9 Department of Clinical Pharmacy and Pharmacotherapeutics, Dubai Pharmacy College for Girls, Dubai, 19099 United Arab Emirates; 10 Department of General Surgery, Mansoura Faculty of Medicine, Mansoura University Hospital, Mansoura, 35516 Egypt; 11 Department of Medicinal Chemistry, College of Pharmacy, University of Sharjah, Sharjah, 27272 United Arab Emirates; 12 Department of Medicinal Chemistry, Faculty of Pharmacy, Mansoura University, Mansoura, 35516 Egypt

**Keywords:** Breast cancer, MCF-7, Quinoline, Anticancer drugs, Raf kinases

## Abstract

**Background:**

Breast cancer is the most common malignancy globally, and is considered a major cause of cancer-related death. Tremendous effort is exerted to identify an optimal anticancer drug with limited side effects. The quinoline derivative RIMHS-Qi-23 had a wide-spectrum antiproliferative activity against various types of cancer cells.

**Methods:**

In the current study, the effect of RIMHS-Qi-23 was tested on MCF-7 breast cancer cell line to evaluate its anticancer efficacy in comparison to the reference compound doxorubicin.

**Results:**

Our data suggest an anti-proliferative effect of RIMHS-Qi-23 on the MCF-7 cell line with superior potency and selectivity compared to doxorubicin. Our mechanistic study suggested that the anti-proliferative effect of RIMHS-Qi-23 against MCF-7 cell line is not through targeted kinase inhibition but through other molecular machinery targeting cell proliferation and senescence such as cyclophlin A, p62, and LC3.

**Conclusion:**

RIMHS-Qi-23 is exerting an anti-proliferative effect that is more potent and selective than doxorubicin.

**Supplementary Information:**

The online version contains supplementary material available at 10.1186/s12885-023-11547-1.

## Introduction

Breast cancer (BC) is the most common malignancy in women worldwide [1]. With 0.5 million fatalities and more than two million new cases in 2020, BC is the second most prominent cause of death in women worldwide [2]. The number of BC new cases is expected to increase to more than three million by 2040, according to the International Agency for Research on Cancer (GLOBOCAN) [3]. According to recent reports, developing countries will suffer from two-thirds of the new breast cancer cases by 2035 [4].

The exact cause of cancer development remains unresolved, but it is hinted that various genetic predispositions, alterations in molecular events including uncontrolled cell proliferation [5], cellular transformations, improper regulation of the cell cycle [6], and angiogenesis, along with increased invasion ensuing metastases [7], are all possible factors. The various cellular and enzymatic pathways responsible for growth and proliferation of cancer cells that have been identified acted as an accelerator for the development of novel anticancer drugs, which increased the available treatment options for patients and consequently, upgraded treatment outcomes [4]. Despite the discovery and the clinical use of many effective anticancer agents, most of them, if not all, suffer from serious drawbacks such as severe side effects. Side effects such as nausea, weight loss, fatigue, loss of appetite, and hair loss stem from non-specific target effects, where anticancer drugs are targeting the normal cells in addition to cancer cells, and affecting pathways unrelated to the progression of cancer [8]. Consequently, the anticancer drug development focuses on hunting safer and more effective drug candidates that exert high selectivity toward cancer cells over normal cells to decrease the severity of side effects and toxicity.

We have previously reported a quinoline-based series possessing an anti-proliferative phenotype as c-Raf kinase inhibitors [9–11]. Among the series, several compounds exhibited broad-spectrum anti-proliferative activity on the NCI-60 cancer cell lines and were more selective toward cancer cells in comparison to the WI-38 normal cell line. Additional in vitro testing, such as investigating possible mechanisms of action (kinase panel as well as caspase-3/7 and lactate dehydrogenase release assays) and pharmacokinetic properties (aqueous solubility, partition coefficient, and Caco-2 A-B permeability), indicated promising results [11]. In the current study, we have opted to test the compound RIMHS-Qi-23 as a potential anti-breast cancer drug using the MCF-7 cell line. This was selected based on its promising in vitro anti-proliferative activity. We also investigated the potential kinase inhibitory effect, molecular mechanisms including cell proliferation, autophagy, and apoptosis as possible mechanisms of action. RIMHS-Qi-23 structure, synthetic procedures, and spectral analysis charts are provided in the Supplementary file.

## Materials and methods

### NCI-60 screening

#### Single-dose testing

**Fig. 1:**
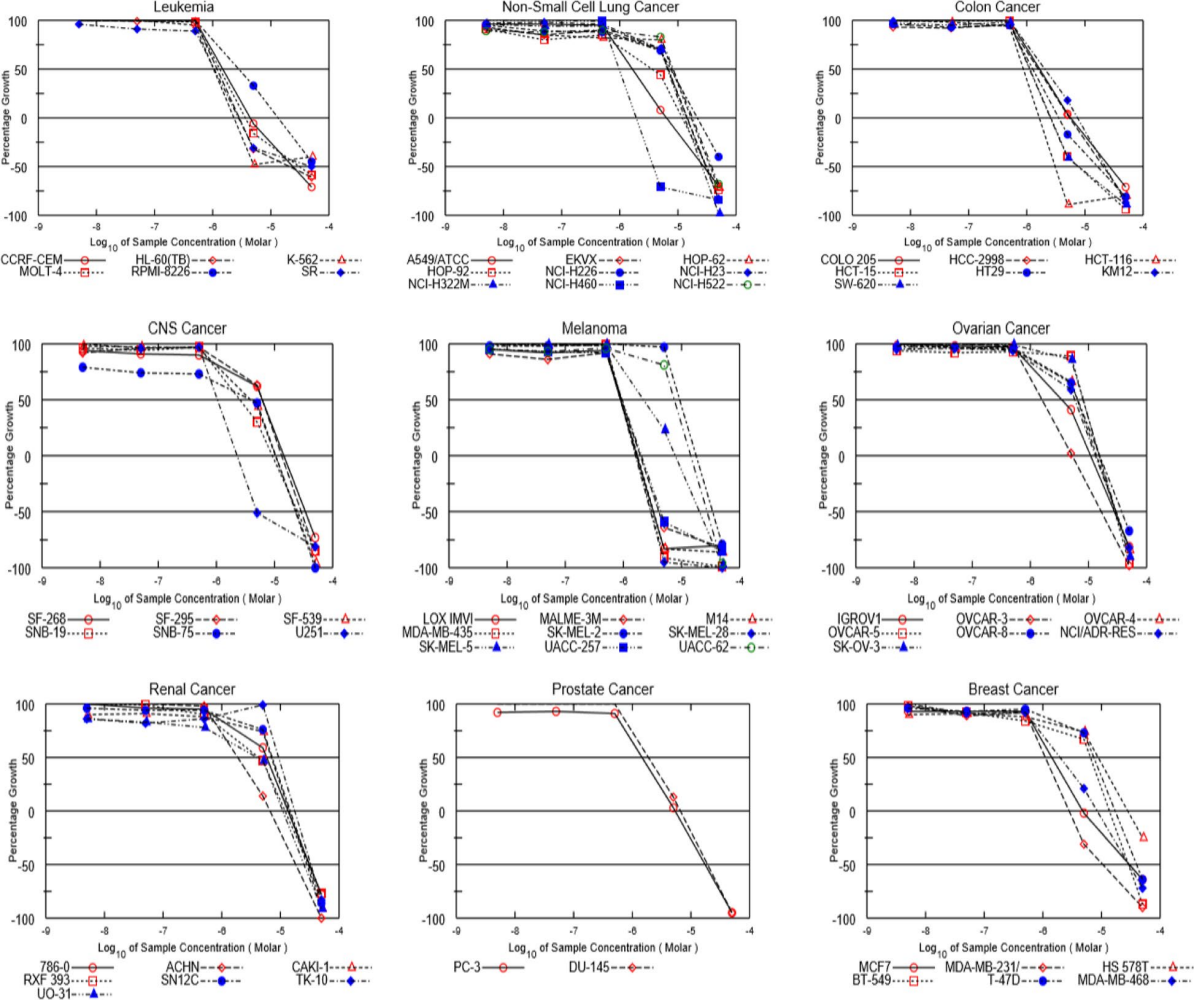
Dose-response curve of RIMHS-Qi-23 against the NCI 60 cell line panel of nine cancer types; from left to right: Leukemia, Non-small cell lung cancer, Colon cancer, CNS cancer, Melanoma, Ovarian cancer, Renal cancer, Prostate cancer and Breast cancer. For breast cancer cell lines panel, the figure shows the effect on MCF-7, MDA-MB-231, H8 578T, BT-549, T-47D and MDA-MB-468

A preliminary in vitro anticancer assay for the target compound was performed against 60 human tumor cell line panel taken from nine different tissues (Fig. 1) in accordance with the protocol of the Drug Evaluation Branch, NCI, Bethesda, MD. To determine the growth inhibition percentages, the target substance was given to the 60 cell lines under study in the single-dose assay at a concentration of 10 µM.

#### Five-dose testing

To assess the potency of RIMHS-Qi-23 and calculate its GI_50_ and TGI values across the 60 cancer cell lines, the compound was tested in a five-dose testing mode. The detailed GI_50_ and TGI values of the compound over each of the tested cell lines for the corresponding cancer type are summarized in Table 1 and Fig. 1.

**Table 1:** GI_50_ and TGI values (µM) of RIMHS-Qi-23 over the NCI-60 cancer cell line panel

Cancer type	Cell line	Tested compound	
		GI_50_^a^	TGI^b^
**Leukemia**	CCRF-CEM	1.48	4.39
	HL-60(TB)	1.18	2.84
	K-562	1.04	2.31
	MOLT-4	1.32	3.65
	RPMI-8226	2.81	13.40
	SR	1.06	2.75
**Non-small cell lung cancer**	A549/ATCC	1.64	6.31
	EKVX	6.70	14.20
	HOP-62	7.86	16.80
	HOP-92	3.58	11.80
	NCI–H226	7.45	21.40
	NCI–H23	6.88	14.50
	NCI–H322 M	6.55	13.00
	NCI–H460	0.975	1.92
	NCI–H522	8.25	17.70
**Colon cancer**	COLO 205	1.72	5.58
	HCC-2998	1.56	5.42
	HCT-116	0.871	1.63
	HCT-15	1.13	2.59
	HT29	1.26	3.52
	KM12	2.04	7.47
	SW-620	1.11	2.54
**CNS cancer**	SF-268	6.13	14.30
	SF-295	6.10	13.20
	SF-539	3.81	10.30
	SNB-19	2.48	9.08
	SNB-75	3.76	10.40
	U251	1.03	2.26
**Melanoma**	LOX IMVI	0.883	1.70
	MALME-3 M	0.931	7.94
	M14	0.933	1.76
	MDA-MB-435	0.907	1.66
	SK-MEL-2	9.21	17.70
	SK-MEL-28	0.889	1.61
	SK-MEL-5	2.19	7.75
	UACC-257	0.951	2.04
	UACC-62	7.48	14.30
**Ovarian cancer**	IGROV1	3.49	10.90
	OVCAR-3	1.59	5.25
	OVCAR-4	6.37	13.70
	OVCAR-5	8.13	15.10
	OVCAR-8	6.47	15.40
	NCI/ADR-RES	5.82	13.20
	SK-OV-3	7.98	15.40
**Renal Cancer**	786–0	5.84	13.50
	ACHN	1.85	6.61
	CAKI-1	6.95	14.00
	RXF 393	4.27	11.90
	SN12C	7.21	14.70
	TK-10	9.29	17.50
	UO-31	4.07	11.00
**Prostate Cancer**	PC-3	1.46	5.37
	DU-145	2.00	6.63
**Breast Cancer**	MCF-7	1.43	4.82
	MDA-MB-231/ATCC	1.09	2.78
	HS 578T	8.77	28.00
	BT-549	6.47	13.70
	T-47D	7.36	17.00
	MDA-MB-468	1.96	8.48

^a^ GI_50_ is the concentration that produces 50% inhibition of the cell growth

^b^ TGI is the concentration that produces 100% inhibition

### Anti-proliferative activity

#### Cell culture

The breast cancer cell line (MCF-7) and normal diploid human fibroblasts (WI-38) were obtained from Medical Experimental Research Center, Faculty of Medicine, Mansoura University and maintained in Dulbecco’s Modified Eagle Medium (DMEM, Sigma-Aldrich), 10% (v/v) Fetal Bovine Serum (FBS, Gibco, Thermo-Fisher, 10270106) and 1% (v/v) Penicillin/Streptomycin (Gibco, Thermo-Fisher, 15140122) are added as supplements. Cells were maintained in incubators at 37 °C with 5% CO_2_ and 95% relative humidity till reaching 80% confluency.

#### MTT assay

To determine the chemotherapeutic impact of RIMHS-Qi-23 on the viability of the MCF-7 cancer cells, 3-(4,5-dimethylthiazol-2-yl)-2,5-diphenyltetrazolium bromide assay (MTT) was performed as described earlier in [12]. Briefly, the cell lines were grown in tissue culture plates at a density of 4 × 10^4^ cells per well for an incubation time of 48 h at 37 °C. The incubated plates were treated either with RIMHS-Qi-23, doxorubicin, or as a negative control with dimethylsulfoxide (DMSO) for 48 h. Then, the culture media was discarded and MTT tetrazolium dye (0.5 mg/mL) was added and incubated at 37 °C for 2 h. The formazan crystals formed were dissolved in DMSO, and the absorbance at 570 nm was measured for each group. For advanced tests, the concentration (IC_50_) needed to reduce viability by 50% was identified using the Formula: Percentage of viable cells = A570 of treated cells/A570 of control cells x 100. This expression measures the impact of different drug concentrations on the proliferation of cells.

### Kinase profiling

RIMHS-Qi-23 was screened using the Kinase HotSpotSM service from Reaction Biology Corp. (http://www.reactionbiology.com). The utilized assay procedure is as reported on Reaction Biology Corp. website (https://www.reactionbiology.com/assay-protocol-hotspot) using 1 µM concentration of ATP.

### Real time quantitative PCR

After checking the results of MTT assay and calculation of IC_50_ values, the experiment was repeated in triplicates of five serial doses concentration of the tested compound as follows: 20 µM, 10 µM, 6.6 µM, 3.3 µM, 1.1 µM versus a triplicate of untreated cells. After 48 h incubation, triplicates of one million cells were collected, immediately homogenized with QIAzol reagent, then RNA and protein extraction (for real-time PCR and western blot, respectively) were immediately started.

 Using the QIAzol reagent (Qiagen, Germany), the total cellular RNA was extracted in accordance with the guidelines given by the manufacturer. Thermo Scientific’s NanoDrop One (USA) was used to measure the amount of RNA present. One µg of RNA was reverse transcribed using the Bioline cDNA synthesis kit (Bioline, USA). A total of 20 µL was used in quantitative real-time PCR (qRT-PCR) [10 µl of HERA SYBR green PCR Master Mix (Willowfort, UK), 1 µl of cDNA template, 2 µl (10 pmol/ µl) of each gene primer and 7 µl of nuclease-free water] utilizing a real-time PCR thermocycler (Azure Cielo 6, Azure, USA). The thermal profile was set at 95 °C for 2 min, followed by 40 cycles of denaturation at 95 °C for 10 s, then annealing and extension at 60 °C for 30 s. Table 2 lists the primer pair sequences that were employed. Glyceraldehyde-3-phosphate dehydrogenase (GAPDH) was used as a control gene. The primer sets were allocated using the Primer 3 software (version 4.1.0), and the Primer- BLAST program (https://www.ncbi.nlm.nih.gov/tools/primer-blast/) was used to evaluate the specificity of the primer sets. An examination of the melting curve of the PCR products was done to determine their specificity. From Vivantis (Vivantis Technologies, Malaysia), primer sets were purchased. The subsequent equation was used to assess the fold change of gene expression using the 2^−ΔΔCT^ method, with ΔCt = Ct target gene - Ct control gene [13].

**Table 2:** The sequence of primers used in qRT-PCR analysis

Gene	Sequence	Product size	RefSeq
**BCL2 associated X, apoptosis regulator (BAX)**	**Forward**: AGCTGCAGAGGATGATTGCC **Reverse**: CCCCAGTTGAAGTTGCCGTC	100 bp	NM_001291428.1
**B-cell lymphoma 2 (BCL2)**	**Forward**: TGTGTGTGGAGAGCGTCAAC **Reverse**: CTACCCAGCCTCCGTTATCC	120 bp	NM_000657.2
**Protein 53 (P53)**	**Forward**: GAGCTGAATGAGGCCTTGGA **Reverse**: CTGAGTCAGGCCCTTCTGTCTT	151 bp	NM_000546.6
**Protein 21 (p21)**	**Forward primer**: GACCAGCATGACAGATTTC **Reverse primer**: TGAGACTAAGGCAGAAGATG	141 bp	NM_000389.5
**Marker Of Proliferation Ki-67 (Ki-67)**	**Forward primer**: AATCCAACTCAAGTAAACGGGG **Reverse primer**: TTGGCTTGCTTCCATCCTCA	127 bp	NM_002417.5
**Glyceraldehyde-3-phosphate dehydrogenase (GAPDH)**	**Forward primer**: CTCTGCTCCTCCTGTTCGAC **Reverse primer**: GCGCCCAATACGACCAAATC	121 bp	NM_002046.7

### Immunoblotting

The total protein extraction from cells was performed by QIAzol reagent (Qiagen, Germany) immediately after obtaining the cells. Protein concentration was measured by Bradford assay (Bosterbio, Canada). Equal amounts of proteins (20 µg) were then separated on SDS-PAGE and transferred to 0.22 mm nitrocellulose membrane (Abcam, USA) using Eco-Line Biometra apparatus (Gottingen, Germany). Page Ruler pre-stained protein ladder 10–180 kDa (ThermoFisher, USA) was used as the molecular size marker. Membranes were then handled as described earlier in [14]. Briefly, the membrane was incubated in 5% non-fat milk for 1 h at room temperature as a blocking agent with gentle shaking. Primary antibodies against LC3B (GeneTex catalog no. GTX127375, dilution 1:1000), p62 (SantaCruz Biotechnology catalog no. sc-48389, dilution 1:500), cyclophilin A (Cell signaling catalog no. 2175, dilution 1:1000) and β-actin (Abcam ab227387, dilution 1:5000) were incubated overnight at 4 °C. After three times of 15 min washing each with TBS-Tween, the membranes were incubated with peroxidase-coupled goat anti-rabbit IgG (Santa Cruz Biotechnology, USA) at 37 °C for 2 h. Membranes were then visualized (after 3 × 15 min washing with TBS-Tween) using chemiluminescent substrate (ClarityTM WesternECL substrate, Catalog no. 1705061, Bio-Rad, USA) and the chemiluminescent signals were collected using ChemiDoc MP imager. Image analysis software was used to quantify the band intensity after normalization to β-actin bands signals.

### Statistical analysis

Using the 2017 release of IBM Corp.‘s SPSS program, data were entered and examined. Armonk, New York: IBM Corp., IBM SPSS Statistics for Windows, Version 25.0. Shapiro-Wilk’s test was used to initially check the normality of quantitative data; if *p* > 0.05, the data were considered to be normally distributed; otherwise, they were not. By looking at boxplots, the existence of significant outliers was tested. When quantitative data were normally distributed, they were reported as mean ± standard error (SE). Six study cell groups’ quantitative data were compared using the One-Way ANOVA test, and then each pair of data were appropriately compared using the post-hoc test. Results are expressed as letters (similar letters = insignificant difference, different letters = significant difference). Tukey adjustment was used as assumption of equal variances were assumed. *p* value < 0.05 was considered statistically significant.

## Results

### RIMHS-Qi-23 shows promising results using NCI-60 cell lines and in comparison, to reference drug doxorubicin

Screening RIMHS-Qi-23 on 60 cancer cell lines showed promising results on single-dose testing mode. It was selected for five-dose testing mode to determine its GI_50_ and TGI values over the 60 cancer cell lines and was reported in comparison to doxorubicin. RIMHS-Qi-23 produced high potency against most of the cell lines (Fig. 1 and Table 1).

### RIMHS-Qi-23 shows higher potency and selectivity using MCF-7 and WI-38 cancer cell lines

**Fig. 2:**
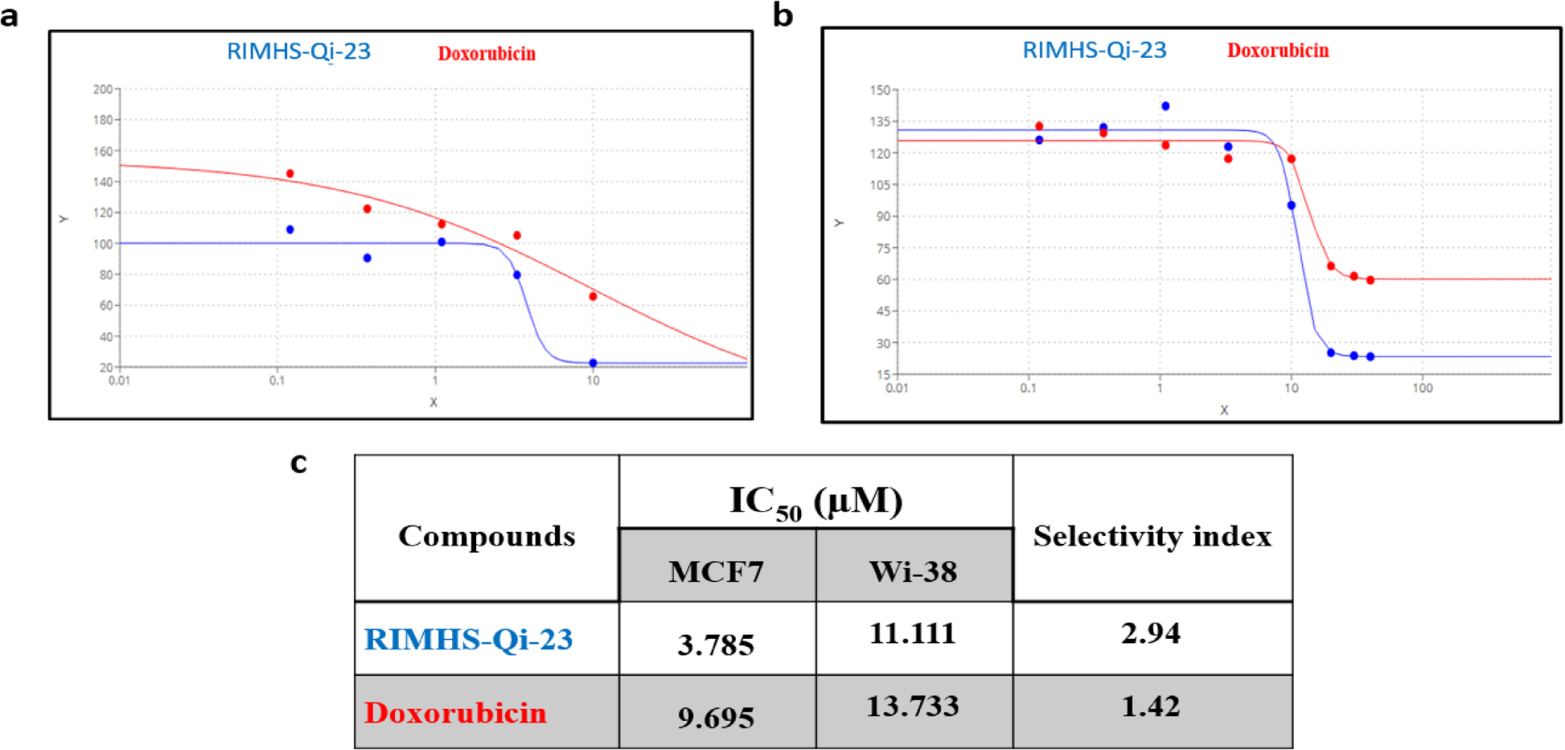
Cytotoxicity assessment of RIMHS-Qi-23 and reference drug doxorubicin using MCF-7 and Wi-38. **a**  Dose response curve showing the cell viability using MCF-7 cell line. **b**  Dose response curve for both drugs using WI-38 cell lines. **c**  IC 50 values of both compounds in MCF-7 and WI-38 cell lines

To evaluate the cytotoxicity of RIMHS-Qi-23, we compared its effect on the widely used MCF-7 breast cancer cell line versus WI-38 normal cells to explore safety and selectivity. RIMHS-Qi-23 was first tested in triplicates of single dose concentration of 10 µM, using doxorubicin as a reference compound, and based on the results of MTT assay, the compound showed higher potency and selectivity. To calculate IC_50_, both drugs were further tested in five serial doses concentrations as follow: 10, 3.3, 1.1, 0.37, and 0.12 µM on both cell lines. However, the concentration of 10 µM failed to reach 50% inhibition in WI-38. Therefore, we opted for three higher concentrations; 20, 30, and 40 µM to draw the dose-response curve and calculate the IC_50_ (Fig. 2).

### RIMHS-Qi-23 did not exhibit inhibitory activity on a panel of 50 kinases

RIMHS-Qi-23 was tested in a single dose at concentration of 1 µM against a panel of 50 kinases to investigate a possible molecular mechanism through kinase inhibition and evaluate off-target activity. Results shown in Table 3, surprisingly show that RIMHS-Qi-23 did not exhibit any sufficient inhibitory activity (more than 50%) against any of the tested kinases. On the contrary, previously reported quinoline derivatives from this series were reported as potent c-Raf inhibitors.

**Table 3:** Residual activity (%) of the panel of 50 kinases after treatment with the RIMHS-Qi-23

Tested kinase	RIMHS-Qi-23 Conc. 1 µM^a^	Tested kinase	RIMHS-Qi-23 Conc. 1 µM^a^
Abl(h)	105	Flt4(h)	101
Abl(T315I)(h)	107	Fms(h)	100
A-Raf(h)	96	GSK3α(h)	110
Aurora-A(h)	107	GSK3β(h)	123
Aurora-B(h)	64	JAK1(h)	110
Aurora-C(h)	113	JAK2(h)	127
B-Raf(h)	91	JAK3(h)	107
B-Raf(V599E)(h)	103	JNK1α1(h)	103
cKit(h)	87	JNK2α2(h)	105
c-Raf(h)	79	JNK3(h)	94
cSRC(h)	108	KDR(h)	102
EGFR(h)	137	MEK1(h)	102
EGFR(L858R)(h)	98	MEK2(h)	113
EGFR(L861Q)(h)	93	MEKK2(h)	104
EGFR(T790M)(h)	129	MEKK3(h)	110
EGFR(T790M,L858R)(h)	104	Met(h)	97
ErbB2(h)	104	mTOR(h)	97
ErbB4(h)	96	PDGFRα(h)	93
FGFR1(h)	110	PDGFRβ(h)	114
FGFR2(h)	105	Pim-1(h)	94
FGFR3(h)	103	Ros(h)	125
FGFR4(h)	93	TrkA(h)	114
Flt1(h)	121	TrkB(h)	117
Flt3(D835Y)(h)	87	TrkC(h)	97
Flt3(h)	80	PI3 Kinase (p120g)(h)	96

^a^ Residual activity of the kinase after treatment with 1 μm of the compound; inhibition percentage = 100-residual activity

### RIMHS-Qi-23 influences cell proliferation and senescence but not cell apoptosis

**Fig. 3:**
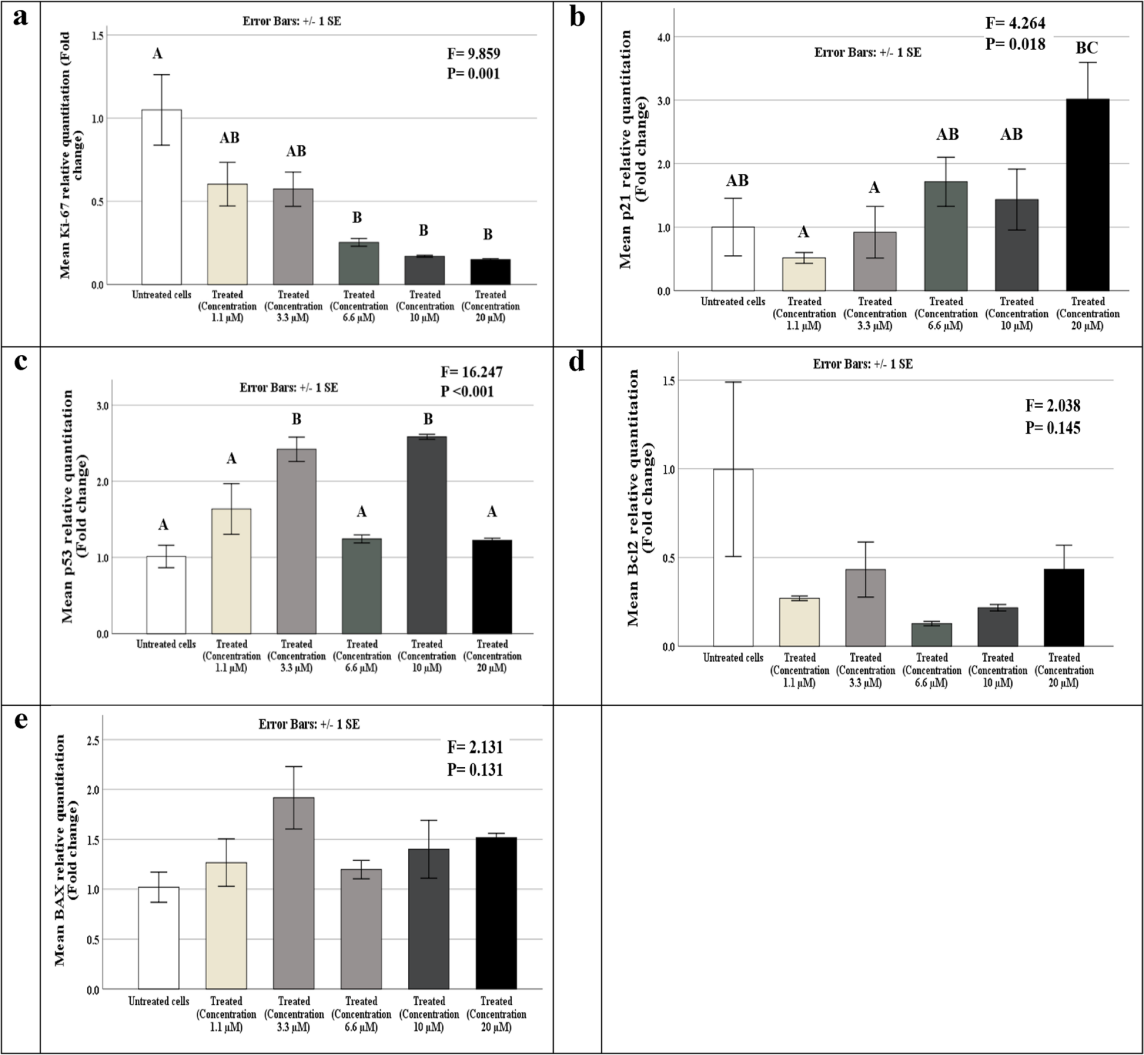
RIMHS-Qi-23 acts through affecting mRNA expression regulation of cell senescence and proliferation genes but not pro or anti apoptosis genes as studied by real-time PCR. **a** Effect of different concentrations (1.1, 3.3, 6.6, 10, 20 µM) on relative expression of Ki-67 mRNA level, showing significant reduction of mRNA expression level **b** p21 **c** p53 d Bcl-2 **e** BAX. Data are represented as mean ± standard error of gene expression fold changes of cells′ triplicates, Capital letters are used to denote *p* values from One-Way ANOVA followed by a post-hoc test (similar letters = a statistically non-significant difference, while different letters = a statistically significant difference). *P* values utilizing Tukey adjustment are bolded to denote significant *p* values (≤ 0.05)

RIMHS-Qi-23’s cytotoxic activity against MCF-7 cell line prompted us to investigate possible mechanism of action through screening the inhibition of genes expression responsible for cell apoptosis; (BAX and BCL2), cell senescence; (p53 and p21) and cell proliferation; Ki67 by real-time PCR. There was a statistically significant difference between the studied concentrations and their effect on p53, p21 and ki-67 genes expression (*p* < 0.001, 0.018 and 0.001, respectively), but RIMHS-Qi-23 treatment did not affect the expression of neither BAX pro-apoptotic gene, nor BCL2 anti-apoptotic genes (*p* = 0.131 and 0.145, respectively), as shown in Fig. 3.

### RIMHS-Qi-23 exhibits its effect through enhancing the autophagy and necrosis pathways

**Fig. 4:**
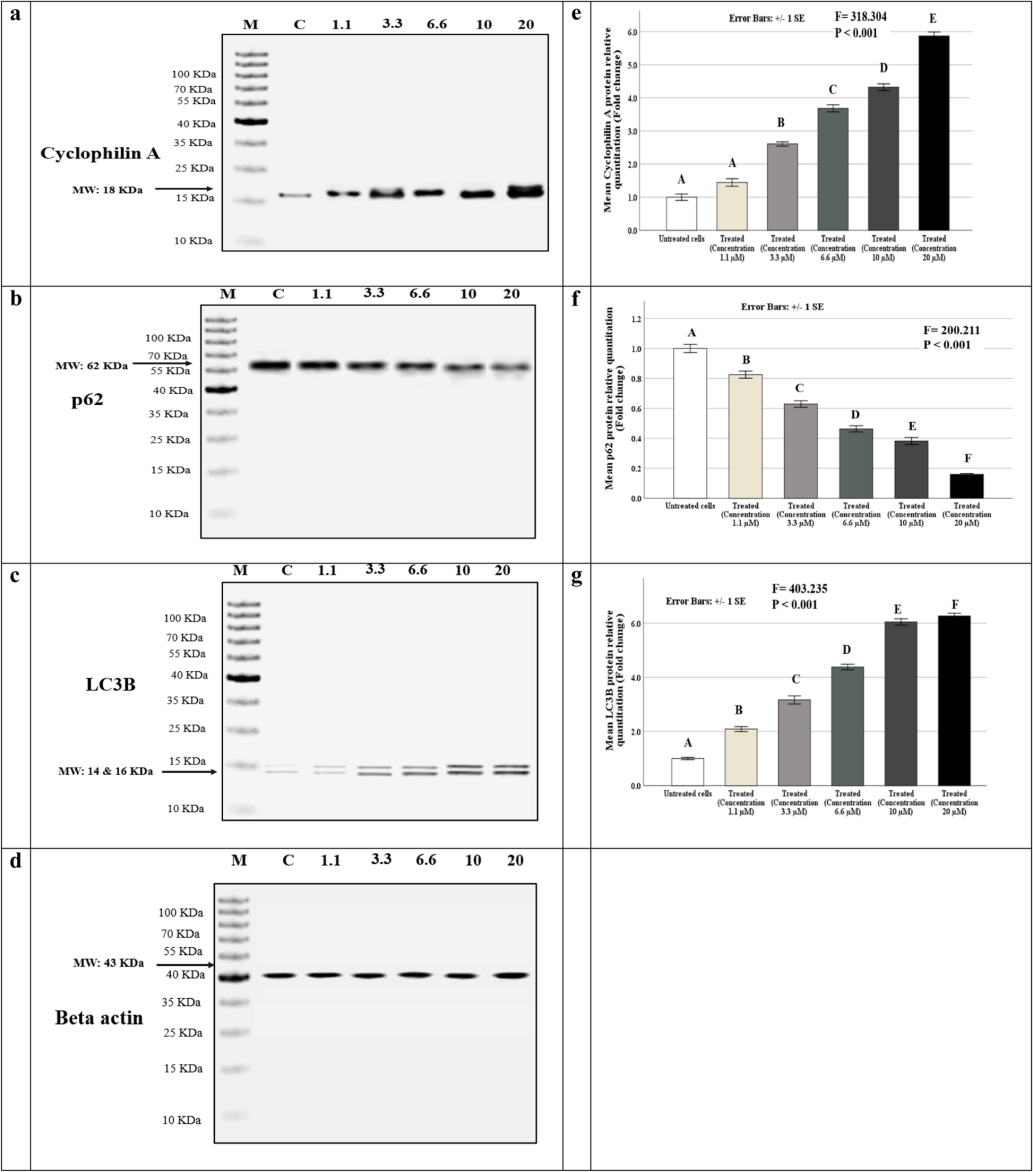
RIMHS-Qi-23 acts through affecting autophagy and necrosis pathways:  **a**-**d**  Immunoblotting of MCF-7 cell lines homogenates following treatment with serial concentration, incubated with cyclophlin A, p62, LC3 and β-actin as loading control **e**-**g**  Quantification of immunoblots band values relative to β-actin were normalized to non-treated cells and represent mean ± SEM. Capital letters represent *p* values from One-Way ANOVA followed by a post-hoc test (similar letters = a statistically non-significant difference, while different letters = a statistically significant difference). Bold values denote significant *p* values (≤ 0.05)

RIMHS-Qi-23’s effect on autophagy pathway and necroptosis pathway was investigated. LC3 and p62 proteins expression were measured to study the effect on autophagy pathway, and cyclophilin A protein expression for necroptosis pathway using western blotting. There was a statistically significant difference between the studied concentrations and their effect on LC3, p62 and cyclophilin A proteins expression (*p* < 0.001 for all). RIMHS-Qi-23 inhibited MCF-7 viability via upregulation of LC3, downregulation of p62, and/or upregulation of the necroptotic cyclophilin A protein expression. All proteins, LC3, p62, and cyclophilin A expressions were highly demonstrated at concentration of 20 µM, but lower at concentration of 10 µM, 6.6 µM, 3.3 µM and 1.1 µM, respectively as shown in Fig. 4.

## Discussion

Breast cancer is the most common malignancy in women, and it is responsible for the highest number of cancer-related deaths in women worldwide [15]. Present therapeutic strategies against breast cancer have different reported side effects including allergies, weight and hair loss, recurrence of cancer, and emergence of drug resistance [16]. Doxorubicin is part of the anthracycline family and is currently considered as the most effective chemotherapeutic drug for breast cancer treatment [17]. However, side effects, drug resistance, and tumor growth result in poor patient prognosis and survival [18]. Collectively, the need to identify a safer, more effective, and more specific alternative is increasing.

In the present study, the compound RIMHS-Qi-23 was selected for cytotoxic studies as an anti-proliferative agent. The data from dose response curve, where RIMHS-Qi-23 was tested on different NCI-60 cell line panel of nine cancer types suggested that it exhibits a promising potent effect against most of cell lines.

Because of the promising results of RIMHS-Qi-23 compound on MCF-7 cancer cell type, and in addition to authors′ interest in this type of cancer (non-invasive & hormone-dependent), a more in-depth study of the effect and mechanism of action of RIMHS-Qi-23 compound was done.

Initial cytotoxicity screening of this compound on the breast cancer cell line MCF-7 displayed significant anticancer activities in a dose- and time- dependent manner. RIMHS-Qi-23 showed a lower IC_50_ than doxorubicin, indicating a greater potency and selectivity (i.e., a higher selectivity index, for MCF-7 cancer cells in comparison to doxorubicin).

To explore the potential mechanism by which RIMHS-Qi-23 is exhibiting its anticancer effect on breast cancer cell line, the compound was tested on a panel of 50 kinases to assess its influence on the activities of different kinases. Although previous quinoline derivatives from the same series exhibited c-Raf inhibitory activity, data did not demonstrate any promising kinase inhibitory effect over the 50 tested kinases. The compound effect was more evident on Aurora-B and c-Raf among others. However, it failed to reach at least a 50% reduction at µM concentration against them. A powerful and broad-spectrum anti-proliferative effect was seen on NCI-60 cell lines by the strongest c-Raf inhibitor in the prior series, which had an IC_50_ of 0.067 µM. The anti-proliferative activity of RIMHS-Qi-23 is comparable and, in some instances, even exhibited superior GI_50_, as shown in Table 2 (NSCLC A549 & NCI-H460, Colon HCC-2998, HCT-116, HT29, & SW-620, CNS U251, Melanoma LOX IMVI, MALME-3 M, M14, MDA-MB-435, SK-MEL-28, & UACC-257, and breast MDA-MB-231) in comparison to the previous series. We believe that RIMHS-Qi-23’s anti-proliferative and kinase activity suggests an off-target effect which is possibly shared with previous quinoline compounds based on high structural similarity it possesses compared with the previous series.

To further investigate the molecular mechanism behind the cytotoxic effect of the drug, the influence of the drug on genes expression levels involved in cancer related signaling pathways, such as apoptosis, cell senescence and cell proliferation were evaluated by qRT-PCR. Five different concentrations ranging from 1.1 to 20 µM were investigated in the current setup. A dose-dependent reduction of cell proliferative Ki-67 mRNA expression suggests that RIMHS-Qi-23 can hinder the proliferation of cancer cells in comparison to healthy cells. Ki-63 is considered as one of the most controversial markers when discussing the treatment decision on breast cancer. Since Ki-67 can be seen during all the cell cycle active phases but is not found in dormant cells, it has become a superb option for measuring the growth fraction of a particular cell population. The ability of RIMHS-Qi-23 to hinder its expression can suggest it as a potential candidate to improve cancer prognosis through inhibition of cancer cell proliferation. To the best of our knowledge, there is no study that has reported the effect of doxorubicin on expression of Ki-67 up to the time of writing this study.

Assessment of the p53/p21 signaling pathway that plays a central role of cellular senescence [19], revealed that RIMHS-Qi-23 treatment showed upregulation of both genes’ mRNA expression levels. Cellular senescence has been considered as a powerful tumor suppressive mechanism [20]. Senescent cells can potentially cause tissue malfunction and/or poor outcomes by secreting pro-inflammatory cytokines that have a detrimental effect on the tissue microenvironment and the nearby cells [21]. Induction of the cellular senescence via p53-dependent pathway is one of the mechanisms which explore the anti-tumor effect of doxorubicin [22]. The effect of treatment on p53/p21 signaling was more evident at higher drug concentrations for p21 in a dose-dependent manner. The effect of the drug on p53 was not very well understood and needs further justification. An initial upregulation was evident at a drug concentration of 3.3 and 10 µM but failed to reach statistical significance in the treated groups at concentration of 6.6 and 20 µM. Upregulation of p53 was reported to be associated with better prognosis via increasing tumor cells sensitivity to the growth inhibitory effect of progesterone [23] suggesting another possible mechanism by which our compound exhibits an anticancer effect on MCF-7 cell line.

Lastly, to evaluate the effect of the compound on the apoptosis pathway, BAX and BCL2 gene expression levels were examined. Our data showed that treatment did not have any influence on their expression levels, which means that the effect of the compound is unlikely to be achieved through regulation of the pro-apoptotic or anti-apoptotic proteins. Our finding contrasts with an earlier report that showed that doxorubicin exhibits its anticancer effect through the reduction of anti-apoptotic genes and elevation of pro-apoptotic genes [24].

Under various stressful circumstances, autophagy is a crucial process in cell recycling and the breakdown of resources for cell homeostasis [25]. Protein light chain 3 (LC3) and p62/SQSTM1 (p62) are associated with autophagosomal membranes that engulf cytoplasmic content for subsequent degradation [26]. Both LC3 and p62 are frequently used as markers to assess autophagy [27]. Treated MCF-7 cells with RIMHS-Qi-23 revealed an increase in LC3 protein expression and reduction of p62, which indicate activated autophagy in cancer cells and inhibited cell proliferation. This substantiates other findings that indicate that autophagy is induced in MCF-7 cells with many anticancer compounds like flavopiridol [28], ursolic acid [29], and baicalein [30]. Moreover, an increased expression of cyclophilin A in the current study suggests that the drug induces the necroptosis pathway as an additional anticancer mechanism. Necroptosis is a controlled form of necrosis in which dead cells burst and release internal substances that may cause an innate immune reaction [31].

## Conclusion

In conclusion, our data suggest that RIMHS-Qi-23 is exerting an anticancer effect, which is more potent and selective than doxorubicin, on the non-invasive, hormone-dependent type of breast cancer (MCF-7). Mechanistic studies have revealed that the compound’s anticancer effect is via a different mechanism apart from its role as a c-Raf kinase inhibitor. The data suggest the involvement of autophagy and necroptosis pathways via regulation of cyclophilin A, p62, p53/p21, and LC3 among others.

## Recommendations

The authors investigated the effect of RIMHS-Qi-23 compound on MCF-7 breast cancer cells being non-invasive type of cancer and hormone-dependent. Further studies will be needed to complement our findings and to further accommodate RIMHS-Qi-23 as a possible anticancer drug candidate, considering its potency and superior selectivity with other standard drugs and performing more extensive research for the possible mechanism(s) of action on several types of cancer cells.

### Supplementary Information

**Additional file 1.**

## Data Availability

The datasets used and/or analyzed during the current study are available from the corresponding author on reasonable request.

## Abbreviations

BAX: BCL2 associated X, apoptosis regulator
BC: Breast cancer
BCL2: B-cell lymphoma 2
cDNA: Complementary DNA
C-RAF: Cellular-rapidly accelerated fibrosarcoma
Ct: Cycle of threshold
DMEM: Dulbecco’s Modified Eagle Medium
FBS: Fetal Bovine Serum
GAPDH: Glyceraldehyde-3-phosphate dehydrogenase
GI_50_: The concentration that produces 50% inhibition of the cell growth
IC50: The concentration needed to reduce viability by 50%
Ki-67: Marker Of Proliferation
LC3: Protein light chain 3
MCF-7: Acronym of Michigan Cancer Foundation-7, referring to the institute where the cell line was established
MTT: 3-(4,5-dimethylthiazol-2-yl)-2,5-diphenyltetrazolium bromide assay
NCI-60: A group of 60 human cancer cell lines used by the National Cancer Institute (NCI)
p21: Protein 21
p53: Protein 53
P62: Protein 62
qRT-PCR: Quantitative real-time PCR
RIHMS-Qi-23: Research Institute for Medical and Health Sciences – Quinoline derivative
RNA: Ribonucleic acid
SE: Standard error
TGI: The concentration that produces 100% inhibition
WI-38: Wistar Institute foetus 38; normal lung fibroblasts
