# Supplementary material for: Anti-proliferative activity of RIHMS-Qi-23 against MCF-7 breast cancer cell line is through inhibition of cell proliferation and senescence but not inhibition of targeted kinases
Source: BMC Cancer. 2023 Nov 2;23:1053. doi: 10.1186/s12885-023-11547-1 (PMC10621201; doi:10.1186/s12885-023-11547-1)

**Anti-proliferative activity of RIHMS-Qi-23 against MCF-7 breast cancer cell line is through inhibition of cell proliferation and senescence but not inhibition of targeted kinases**

**Randa El-Gamal ^1,2,3*^, Sara Elfarrash ^2,4^, Mohammad EL-Nablaway ^1,5^, Asmaa Ahmed Salem ^6^, Seyed-Omar Zaraei** **^7^**, **Hanan S. Anbar** **^8^**, **Ashraf Shoma ^9^, Mohammed I. El-Gamal ^7,10,11*^**

**^1^** Department of Medical Biochemistry & Molecular Biology, Faculty of Medicine, Mansoura University, 35516, Mansoura, Egypt

**^2^** Medical Experimental Research Center (MERC), Faculty of Medicine, Mansoura University, 35516, Mansoura, Egypt

**^3^** Department of Medical Biochemistry, Faculty of Medicine, Horus University, New Damietta, Egypt

**^4^** Department of Medical physiology, Faculty of Medicine, Mansoura University, 35516, Mansoura, Egypt

**^5^** Department of Basic Medical Sciences, College of Medicine, AlMaarefa University, PO Box 71666, Riydah 11597, Kingdom of Saudi Arabia

**^6^** Department of Clinical Oncology and Nuclear Medicine, Faculty of Medicine, Mansoura University, Egypt

**^7^** Research Institute of Medical and Health Sciences, University of Sharjah, Sharjah 27272, United Arab Emirates

**^8^** Department of Clinical Pharmacy and Pharmacotherapeutics, Dubai Pharmacy College for Girls, Dubai, 19099, United Arab Emirates

**^9^** Department of General Surgery, Mansoura Faculty of Medicine, Mansoura University Hospital, Mansoura, 35516, Egypt

**^10^** Department of Medicinal Chemistry, College of Pharmacy, University of Sharjah, Sharjah 27272, United Arab Emirates

**^11^** Department of Medicinal Chemistry, Faculty of Pharmacy, Mansoura University, Mansoura 35516, Egypt

*** Corresponding author:**

- **Randa El-Gamal**

Postal address: Arab Republic of Egypt, Al-Daqahlia Governorate, Mansoura, University of Mansoura, Faculty of Medicine, Department of Medical Biochemistry, Postal office number 35516.

ORCID: 0000-0003-0211-1030

E-mail address: [drrandaelgamal@mans.edu.eg](mailto:drrandaelgamal@mans.edu.eg), [drrandaelgamal@yahoo.com](mailto:drrandaelgamal@yahoo.com)

Tel. No.: 002-01007107188

- **Mohammed I. El-Gamal**

E-mail address: [drmelgamal2002@gmail.com](mailto:drmelgamal2002@gmail.com); [malgamal@sharjah.ac.ae](mailto:malgamal@sharjah.ac.ae); [drmelgamal@mans.edu.eg](mailto:drmelgamal@mans.edu.eg)

| **Content** | **Page No.** |
| --- | --- |
| Synthetic procedures utilized to synthesize **RIHMS-Qi-23** | S3-S7 |
| NMR and LC-MS charts of **RIHMS-Qi-23** | S8-S10 |

# **Synthetic procedures**

**Scheme 1.** Reagents and condition: a) Fe/HCl, EtOH/H_2_O, rt, overnight, 85%; b) K_2_CO_3_, acetone, reflux, 5 h, 90%; c) piperidine, EtOH, reflux, 24 h, 88%; d) acetic acid, HCl, 95 °C, 16 h, 75%; e) K_2_CO_3_, DMF, rt, overnight, 65%; f) Pd/C, H_2_, THF, rt, 2 h, 60%; g) 3,5-bis(trifluoromethyl)benzoic acid, HOBt, EDCI, TEA, rt, 12 h, 64%.

## General

The synthesized compounds were analyzed by ^1^H NMR and ^13^C NMR using a Bruker Avance (500 MHz) spectrometer. LC-MS analysis was done using LC-MS analyzer (Waters Corporation, MA, USA). The melting point was measured on a Stuart melting point apparatus (Staffordshire, UK), and are uncorrected. All the solvents and reagents were obtained from commercial companies and used as is with no prior purification. The final and intermediate compounds were purified by column chromatography (silica gel, 230-400 mesh) using laboratory reagent grade solvents.

## Synthesis of 2-amino-4-(benzyloxy)-5-methoxybenzaldehyde (3)

To a solution of 4-(benzyloxy)-5-methoxy-2-nitrobenzaldehyde (**2**, 288 mg, 1.0 mmol) in ethanol (6 mL), water (1.5 mL) was added. After that, iron powder (335 mg, 6 mmol) and Conc. HCl (0.1 mL) were added to the reaction mixture. It was allowed to stir at room temperature overnight. The mixture was filtered, and the filtrate was concentrated in vacuo, water (5 mL) was added then extracted with ethyl acetate (3 x 10 mL). The organic layer extract was dried by anhydrous sodium sulfate and evaporated to dryness. The product was purified by crystallization from hexane and ethyl acetate. Yield: 85%; ^1^H NMR (CDCl_3_, 500 MHz) δ 9.62 (s, 1H, CHO), 7.36-7.24 (m, 6H), 6.85 (s, 1H), 6.06 (s, 1H), 5.94 (brs, 1H) [Ar-H, NH_2_], 5.09 (s, 2H, Ph-CH_2_-O), 3.78 (s, 3H, OCH_3_); LC-MS: 258.21 [M + 1] ^+^.

## Synthesis of 1-(4-nitrophenoxy)propan-2-one (6)

A mixture of 4-nitrophenol (1.0 g, 5.12 mmol) or 4-nitrothiophenol (1.08 g, 5.12 mmol), chloroacetone (0.474 g, 5.12 mmol), and potassium carbonate (0.707 g, 5.12 mmol) in anhydrous acetone (15 mL) was heated under reflux for 5 h. The reaction mixture was filtered, and the solid residue was washed with acetone (3 x 10 mL). The combined filtrate and wash were evaporated under reduced pressure. The residue was washed and crystallized from hexanes to get the pure crystalline title compounds. Yield 90%. ^1^H NMR (CDCl_3_, 300 MHz) δ 8.17 (d, *J* = 9.0 Hz, 2H), 7.40 (d, *J* = 9.0 Hz, 2H), 3.86 (s, 2H), 2.37 (s, 3H).

## Synthesis of 3-(4-nitrophenoxy)-7-(benzyloxy)-6-methoxy-2 methylquinoline (7)

A mixture of compound **3** (257 mg, 1.0 mmol), compound **6** (195 mg, 1.0 mmol), and piperidine (0.119 g, 1.4 mmol) in ethanol (10 mL) was heated under reflux for 24 hours. After completion of the reaction, it was allowed to cool to room temperature then to 0 °C to obtain the product crystallized out. The product was filtered, washed with ethanol (3 mL), and dried.

Yield: 88%; ^1^H NMR (CDCl_3_, 500 MHz) δ 8.24 (d, 2H, *J* = 3.5 Hz), 7.59 (s, 1H), 7.50 (d, 2H, *J* = 7.5 Hz), 7.44-7.38 (m, 3H), 7.34 (d, 1H, *J* = 7.5 Hz), 7.02-6.98 (m, 3H) [Ar-H], 5.31 (s, 2H, Ph-CH_2_-O), 3.99 (s, 3H, OCH_3_), 2.53 (s, 3H, quinoline-CH_3_); LC-MS: 417.21 [M + 1] ^+^.

## Synthesis of 3-(4-nitrophenoxy)-6-methoxy-2-methylquinolin-7-ol (8)

Compound **7** (416 mg, 1.0 mmol) was dissolved in a mixture of glacial acetic acid (3 mL) and Conc. HCl (5 mL). The mixture was heated at 95 °C for 16 h. The reaction mixture was cooled to rt, concentrated under vacuum, and separated between ethyl acetate (2 x 10 mL) and saturated aqueous potassium carbonate. The organic layer was washed with saline (3 x 5 mL), dried using anhydrous sodium sulfate, and evaporated to dryness. The product was purified by column chromatography using hexane and ethyl acetate.

Yield: 75%; ^1^H NMR (CDCl_3_, 500 MHz) δ 11.49 (brs, 1H, OH), 8.51 (s, 1H), 8.30 (d, 2H, *J* = 9.5 Hz), 7.71 (s, 1H), 7.52 (s, 1H), 7.36 (d, 2H, *J* = 9.5 Hz) [Ar-H], 3.92 (s, 3H, OCH_3_), 2.69 (s, 3H, quinoline-CH_3_); ^13^C NMR (CDCl_3_, 125 MHz) δ 163.1, 151.5, 148.9, 148.1, 145.8, 143.1, 126.3, 124.5, 123.1, 116.6, 110.7, 104.2 [Ar-C], 56.3 (OCH_3_), 20.2 (quinoline-CH_3_); LC-MS: 312.11 [M + 1]^+^.

## Synthesis of 7-(2-(piperidin-1-yl)ethoxy)-3-(4-nitrophenoxy)-6-methoxy-2-methylquinoline (10)

To a solution of compound **8** (326 mg, 1.0 mmol) is dry DMF (3 mL), anhydrous potassium carbonate (414 mg, 3.0 mmol) was added. The mixture was stirred at room temperature for 10 min, then 1-(2-chloroethyl)piperidine hydrochloride (**9**, 275 mg, 1.5 mmol) was added thereto. The reaction mixture was allowed to stir at room temperature overnight. After reaction completion, saline (10 mL) was added to the reaction mixture and the product was extracted using ethyl acetate (3 x 10 mL). The organic extract was washed with saline (15 mL) and then dried with anhydrous sodium sulfate. The solvent was evaporated in vacuo and the product was purified by normal phase column chromatography using hexane/ethyl acetate.

Yield: 65%; ^1^H NMR (CDCl_3_, 500 MHz) δ 8.22 (q, 2H, *J* = 2.0 Hz), 7.58 (s, 1H), 7.40 (s, 1H), 7.00 (q, 2H, *J* = 2.0 Hz), 6.95 (s, 1H) [Ar-H], 4.34 (t, 2H, *J* = 6.0 Hz, NCH_2_CH_2_O), 3.96 (s, 3H, OCH_3_), 2.96 (t, 2H, *J* = 6.5 Hz, NCH_2_CH_2_O), 2.61 (brs, 4H, piperidine CH_2_-N-CH_2_), 2.54 (s, 3H, quinoline-CH_3_), 1.65 (t, 4H, *J* = 5.0 Hz, piperidine CH_2_-CH_2_-N-CH_2_-CH_2_), 1.48 (d, 2H, *J* =4.5 Hz, piperidine N-CH_2_-CH_2_-CH_2_); ^13^C NMR (CDCl_3_, 125 MHz) δ 163.1, 151.6, 151.0, 150.5, 146.0, 143.0, 142.8, 126.3, 124.2, 123.4, 116.6, 108.8, 104.8 [Ar-C], 66.9 (NCH_2_CH_2_O), 57.4 (OCH_3_), 56.2 (NCH_2_CH_2_O), 55.1 (piperidine CH_2_-N-CH_2_), 25.9 (piperidine CH_2_-CH_2_-N-CH_2_-CH_2_), 24.2 (piperidine N-CH_2_-CH_2_-CH_2_), 20.2 (quinoline-CH_3_).

## Synthesis of 4-(7-(2-(piperidin-1-yl)ethoxy)-6-methoxy-2-methylquinolin-3-yloxy)benzenamine (11)

A mixture of compound **10** (437 mg, 1.0 mmol) and palladium over charcoal (5%) in THF (15 mL) was stirred in hydrogen atmosphere at room temperature for 2 h. The reaction mixture was then filtered through celite, and the filtrate was evaporated to dryness under vacuum to get the title product. They were used in the next reactions as such without any purification.

## Synthesis of N-(4-(7-(2-(Piperidin-1-yl)ethoxy)-6-methoxy-2-methylquinolin-3-yloxy)phenyl)-3,5-bis(trifluoromethyl)benzamide (1, RIMHS-Qi-23)

A mixture of compound **11** (73 mg, 0.18 mmol), appropriate benzoic acid derivative (0.36 mmol), HOBt (54 mg, 0.39 mmol), and EDCI (87 mg, 0.45 mmol) in dry DMF (2 mL) was cooled to 0 ^°^C under nitrogen atmosphere. Triethylamine (0.06 mL, 0.45 mmol) was added thereto at 0 ^°^C. The mixture was then stirred at room temperature for 12 h. The reaction mixture was partitioned between water (10 mL) and ethyl acetate (10 mL), and the organic layer was separated. The aqueous layer was then extracted with ethyl acetate (3 X 5 mL), and the combined organic extracts were washed with saline and dried with anhydrous Na_2_SO_4_. The organic solvent was evaporated, and the crude residue was purified by normal phase column chromatography using hexane-ethyl acetate to get the target product.

Yield: 64%; mp: 66-69 °C; ^1^H NMR (DMSO-*d_6_*, 500 MHz) δ 8.59 (brs, 1H), 8.50 (brs, 2H), 8.23 (brs, 1H, NH), 8.00 (brs, 1H), 7.80 (d, 2H, *J* = 9.0 Hz), 7.58 (s, 1H), 7.38 (s, 1H), 7.11-7.09 (m, 2H) [Ar-H], 4.47 (t, 2H, *J* = 5.5 Hz NCH_2_CH_2_O), 3.95 (d, 3H, *J* = 6.0 Hz, OCH_3_), 3.43 (t, 2H, *J* = 5.0 Hz, NCH_2_CH_2_O), 3.20 (brs, 4H, piperidine CH_2_-N-CH_2_), 2.66 (s, 3H, quinoline-CH_3_), 1.96 (s, 4H, piperidine CH_2_-CH_2_-N-CH_2_-CH_2_), 1.66 (brs, 2H, piperidine N-CH_2_-CH_2_-CH_2_); ^13^C NMR (DMSO-*d_6_*, 125 MHz) δ 155.4 (CO), 153.2, 151.7, 151.6, 151.5, 151.0, 141.3, 137.6, 136.6, 125.7, 125.6, 125.4, 122.7, 121.3, 120.5, 118.3, 118.1, 108.7, 106.4 [Ar-C], 66.7 (NCH_2_CH_2_O), 58.1 (OCH_3_), 56.5 (NCH_2_CH_2_O), 55.8 (piperidine CH_2_-N-CH_2_), 25.8 (piperidine CH_2_-CH_2_-N-CH_2_-CH_2_), 24.3 (piperidine N-CH_2_-CH_2_-CH_2_), 19.7 (quinoline-CH_3_); LC-MS: 648.17 [M + 1]^+^; CHN analysis: calculated C:61.20%, H:4.82%, N:6.49%; found: C:61.10%, H:4.75%, N:6.60%.

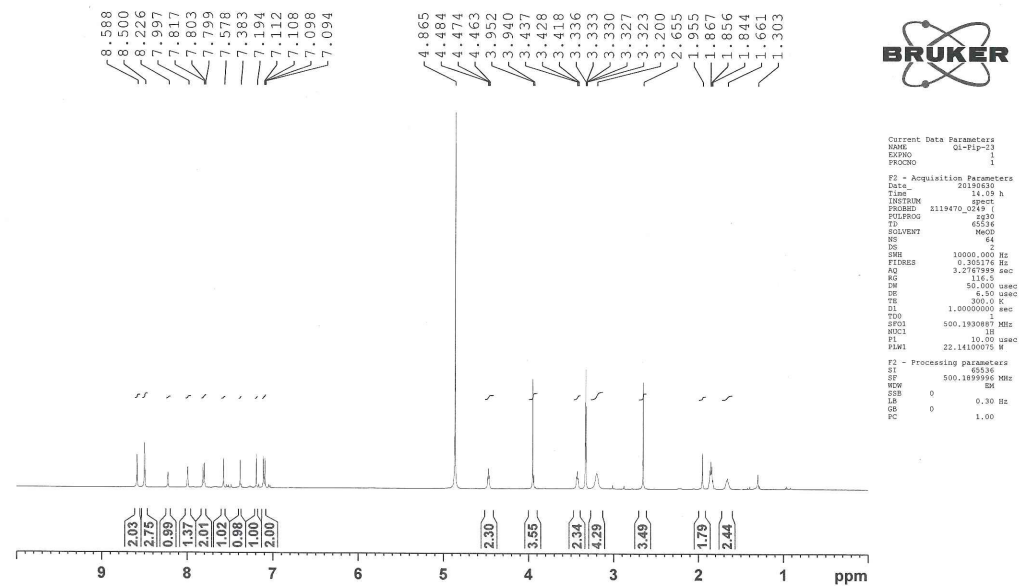

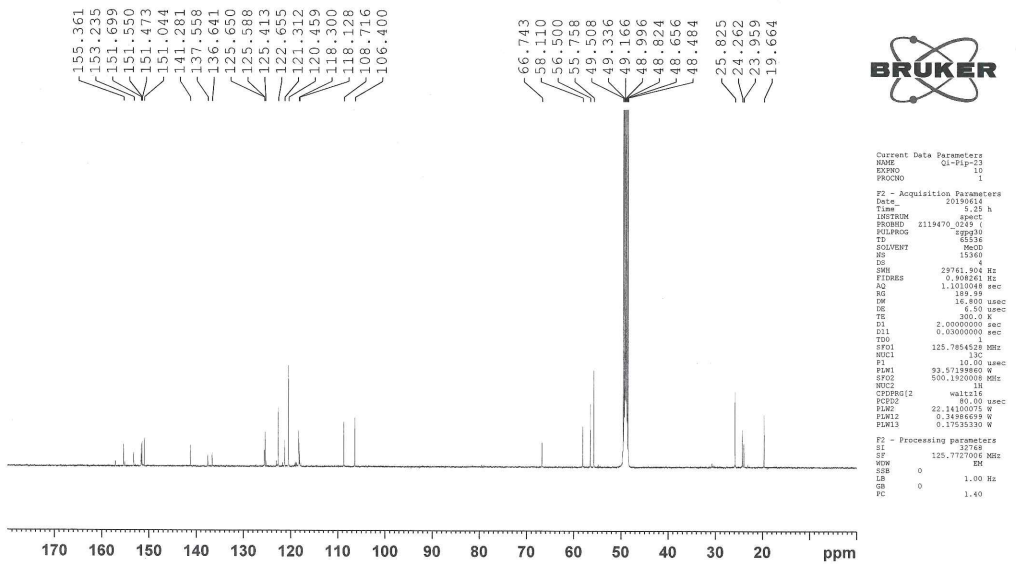

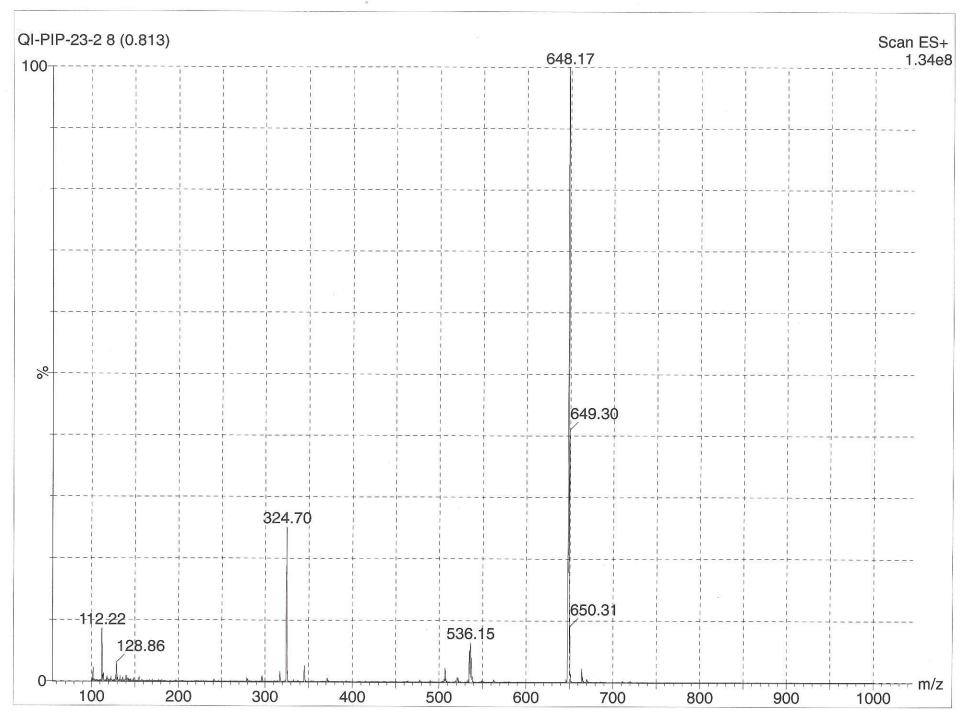

Supplement: Supplementary file 1 — Additional file 1. [file 12885_2023_11547_MOESM1_ESM.docx]
